# Subtype-MGTP: a cancer subtype identification framework based on multi-omics translation

**DOI:** 10.1093/bioinformatics/btae360

**Published:** 2024-06-10

**Authors:** Minzhu Xie, Yabin Kuang, Mengyun Song, Ergude Bao

**Affiliations:** College of Information Science and Engineering, Hunan Normal University, Changsha 410081, China; Key Laboratory of Computing and Stochastic Mathematics (Ministry of Education), Changsha 410081, China; College of Mathematics and Statistics, Hunan Normal University, Changsha 410081, China; College of Information Science and Engineering, Hunan Normal University, Changsha 410081, China; College of Information Science and Engineering, Hunan Normal University, Changsha 410081, China; School of Software Engineering, Beijing Jiaotong University, Beijing 100044, China

## Abstract

**Motivation:**

The identification of cancer subtypes plays a crucial role in cancer research and treatment. With the rapid development of high-throughput sequencing technologies, there has been an exponential accumulation of cancer multi-omics data. Integrating multi-omics data has emerged as a cost-effective and efficient strategy for cancer subtyping. While current methods primarily rely on genomics data, protein expression data offers a closer representation of phenotype. Therefore, integrating protein expression data holds promise for enhancing subtyping accuracy. However, the scarcity of protein expression data compared to genomics data presents a challenge in its direct incorporation into existing methods. Moreover, striking a balance between omics-specific learning and cross-omics learning remains a prevalent challenge in current multi-omics integration methods.

**Results:**

We introduce Subtype-MGTP, a novel cancer subtyping framework based on the translation of Multiple Genomics To Proteomics. Subtype-MGTP comprises two modules: a translation module, which leverages available protein data to translate multi-type genomics data into predicted protein expression data, and an improved deep subspace clustering module, which integrates contrastive learning to cluster the predicted protein data, yielding refined subtyping results. Extensive experiments conducted on benchmark datasets demonstrate that Subtype-MGTP outperforms nine state-of-the-art cancer subtyping methods. The interpretability of clustering results is further supported by the clinical and survival analysis. Subtype-MGTP also exhibits strong robustness against varying rates of missing protein data and demonstrates distinct advantages in integrating multi-omics data with imbalanced multi-omics data.

**Availability and implementation:**

The code and results are available at https://github.com/kybinn/Subtype-MGTP.

## 1 Introduction

Cancer is widely recognized as a complicated disease, presenting a significant challenge to targeted therapies (Bianchini *et al.* 2016). The identification of cancer subtypes offers valuable insights into understanding the heterogeneity of tumors (Sims *et al.* 2007). By categorizing patients into different clinical subgroups, cancer subtyping enables researchers to explore the pathogenesis of the disease and develop personalized treatment strategies (Zhao *et al.* 2019). Traditional cancer subtyping approaches are mainly based on specific gene (or protein) expression levels or gene mutations (Kittaneh *et al.* 2013, Zhang *et al.* 2013). However, with the rapid development of high-throughput sequencing technologies, numerous cancer projects have accumulated vast repositories of multi-omics data from cancer patients (Lightbody *et al.* 2019). Consequently, the utilization of multi-omics data for cancer subtyping has gained significant attention in the field.

In the past decade, various computational methods have been developed to integrate multi-omics data to identify cancer subtypes. These methods can be broadly categorized into supervised and unsupervised approaches. Supervised methods, reliant on sample labels, may be limited in their ability to uncover novel subtypes. Consequently, the prevailing trend in current methods favors unsupervised strategies. Using clustering-based techniques, these methods evaluate sample similarity to partition patients into distinct subgroups. Initially, they mainly adopted either feature concatenation-based or ensemble-based strategies (Tini *et al.* 2019). Feature concatenation-based methods typically concatenate multi-omics data into a unified comprehensive feature matrix, subsequently undergoing clustering via established algorithms [e.g. K-means (Ding and He 2004)]. However, this simplistic concatenation overlooks distribution differences within multi-omics data and may disproportionately emphasize omics data with a greater number of features. Addressing this challenge involves performing feature normalization and selection. Notably, to deal with the high dimensionality of multi-omics data, regularization techniques are often used to mitigate the risk of overfitting. For instance, by incorporating nuclear norm regularization, LRAcluster (Wu *et al.* 2015) uses low-rank approximation to derive a common latent representation across different data types, which is subsequently clustered using K-means. Ensemble-based clustering methods allow the clustering of individual omics data using different algorithms, and then combine the outcomes into a unified result (Rappoport and Shamir 2018). As a representative ensemble clustering method, PINS (Nguyen *et al.* 2017) integrates the clustering outcomes by analyzing the connectivity matrices derived from various types of omics data.

Obviously, the feature concatenation-based methods excel in preserving the maximum information from each omics dataset, while the ensemble-based methods leverage the most suitable algorithms to achieve optimal clustering results for individual omics data types. However, both of them may overlook inter-omics correlations. Consequently, significant efforts have been dedicated to exploring interactions among various types of omics data. For instance, MCCA (Witten and Tibshirani 2009) identifies sparse linear combinations and maximizes correlations among them, enabling the linear projection of multi-omics data into lower-dimensional spaces for clustering. Similarly, iCluster (Shen *et al.* 2009), as a joint latent variable model, incorporates sparsity-inducing penalties or regularization terms to encourage the selection of a sparse set of features that contribute significantly to the clustering structure. However, both MCCA and iCluster require feature pre-screening, which may lead to the loss of crucial biological signals. In contrast, network-based methods have shown effectiveness in noise reduction and similarity reinforcement. These methods construct sample similarity networks for each omics data type, and use network fusion strategies such as SNF (Wang *et al.* 2014) and NEMO (Rappoport and Shamir 2019) for cross-omics correlation learning. However, these methods are constrained by predefined similarity measurements.

In recent years, there has been a surge in the development of deep learning models for cancer subtyping, leveraging the powerful feature extraction capabilities of neural networks to achieve impressive performance. Notably, Subtype-GAN (Yang *et al.* 2021) uses a multiple-input-multiple-output neural network to accurately capture intrinsic representations of omics data, followed by consensus clustering with a Gaussian mixture model to identify molecular subtypes of tumor samples. Similarly, two novel end-to-end multi-omics cancer subtyping models Subtype-DCC (Zhao *et al.* 2023) and DMCL (Chen *et al.* 2023a) optimize representation learning and clustering jointly. MOCSS (Chen *et al.* 2023b) integrated improved contrastive learning techniques and orthogonality constraints to fully explore both omics-specific learning and cross-omics correlation learning.

Despite previous studies have made significant progress in cancer subtyping, several challenges remain unresolved. Firstly, owing to the abundant genomic data, most cancer subtyping methods primarily integrate multi-omics data at the genomic level. However, there exists a notable gap between genotype and phenotype, which could potentially compromise subtyping performance. Secondly, striking a delicate balance between omics-specific learning and cross-omics learning during data integration poses a formidable challenge. An excessive focus on either aspect may lead to insufficient exploitation of the complementary information, thereby hampering overall performance.

This study endeavors to integrate proteomics into cancer subtyping, recognizing its closer relevance to the phenotype compared to genomics. However, a significant challenge arises from the limited availability of protein expression data relative to genomics data in the TCGA database, with some patients lacking such data. This scarcity presents a substantial obstacle in utilizing proteomics data for cancer subtyping. Moreover, even with sufficient protein expression data, achieving a balance between omics-specific learning and cross-omics learning remains a challenge when directly integrating it with other multi-omics datasets. To tackle these challenges, we propose a two-stage cancer subtyping framework named Subtype-MGTP. Subtype-MGTP aims to bridge the genotype-to-phenotype gap while circumventing conflicts between omics-specific learning and cross-omics learning. In the first stage, Subtype-MGTP uses a translation module to predict protein expression levels for all patients based on their multiple genomics data guided by partially available protein expression data. In the second stage, a deep subspace contrastive clustering module is deployed to subtype patients based on the predicted protein expression data. We benchmarked Subtype-MGTP against nine other state-of-the-art clustering methods. Extensive experiments conducted on eight widely used cancer datasets demonstrated the superiority of our model. In addition, we conducted a case study on specific datasets to illustrate the biological significance of clustering results.

In summary, the main highlights of Subtype-MGTP can be outlined as follows:

- Enhanced efficiency in genomics data mining, leveraging protein data that closely correlates with the phenotype.
- Skillful circumvention of omics-specific learning and cross-omics learning balance issue inherent in multi-omics data integration via the translation of multi-omics data into single-omics data.
- Improved performance in traditional subspace clustering through the integration of graph neural networks and contrastive learning, facilitating the discovery of meaningful cancer subtypes.
- Enhanced interpretability of clustering results, as demonstrated by a case study showcasing biologically distinguishable subtypes.

## 2 Materials and methods

**Figure 1.**
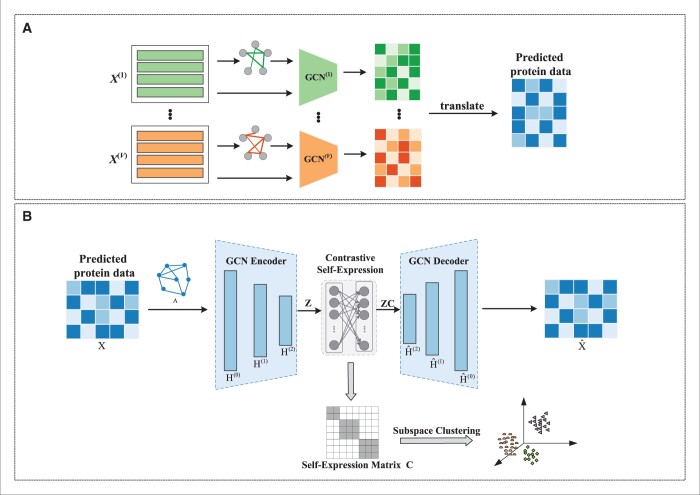
The framework of Subtype-MGTP. Subtype-MGTP consists of two modules: (A) multi-omics data translation module. This module takes multiple genomics data as input. Through the feature extracting of the corresponding Graph Convolutional Network (GCN), a low-dimensional representation for each omics is obtained, which is then translated into predicted protein data. (B) Deep subspace contrastive clustering module. This module takes the predicted protein data as input. Z denotes the input of the contrastive self-expression layer, extracted by the encoder. ZC denotes the output of the contrastive self-expression layer, and serves as the input of the decoder. Notably, the self-expression coefficient matrix C is the key element used for the subsequent clustering task.

As presented in Fig. 1, Subtype-MGTP consists of two components. Firstly, a multi-omics data translation module, guided by protein data, aims to convert multi-type genomics data into proteomics data. Secondly, leveraging the translated proteomics data, a cancer subtype identification module applies improved subspace clustering to assign subtype labels to each sample.

### 2.1 Benchmark datasets

In this study, we utilized eight TCGA datasets representing cancers BLCA, BRCA, KIRC, LUAD, SKCM, STAD, UCEC, and UVM to assess the performance of Subtype-MGTP. The datasets were downloaded via the TCPA portal (Li *et al.* 2013). Each of these datasets includes five types of omics data: mRNA, miRNA, Copy Number (CN), DNA methylation, and protein expression. The total datasets comprise 3851 cancer patient samples (Yang *et al.* 2021), and each sample has four types of genomics data (mRNA, miRNA, CN, and DNA methylation). Specifically, each sample is characterized by 3217 mRNA features, 3105 copy number features, 383 miRNA features, and 3139 DNA methylation features. However, only 3025 of the 3851 samples have protein expression data. After performing data preprocessing, such as removing missing values and standardization, we obtained the normalized expression levels of 455 proteins for each sample. More details about the datasets can be found in Table 1.

**Table 1.** The statistics of the datasets.^a^

	BLCA	BRCA	KIRC	LUAD	SKCM	STAD	UCEC	UVM	ALL
Genomics	399	1031	488	490	446	407	510	80	3851
Proteomics	331	829	438	346	333	329	407	12	3025

a Each element denotes the number of samples in the cancer dataset that possess corresponding omics data.

### 2.2 Multi-omics data translation module

Since not all samples have available protein data, integrating protein data into existing multi-omics clustering models presents a challenge. In addressing this issue and striving to mitigate any potential imbalance between omics-specific learning and cross-omics learning, we explore the integration of protein data through translation methods. This section introduces a novel translation module designed to convert multiple genomics datasets into proteomics data, leveraging graph convolutional neural networks (GCNs) for enhanced accuracy and efficiency.

The multi-omics data of cancer patients often exhibit intricate interrelationships. GCNs offer a significant advantage over traditional neural networks, as they can effectively capture graph structure information by propagating and aggregating features between nodes through convolutional operations. At first, a patient-to-patient similarity graph is constructed for each omics data type, incorporating the feature matrix *X* of samples and the node adjacency matrix *A*. The element *A_ij_* in the adjacency matrix *A* represents the similarity between patient *i* and patient *j*, calculated as follows:

(1) $$\begin{array}{c} A_{ij}=\frac{s(i,j)}{\sum_{r\in \eta_{i}}s(i,r)}\cdot I(j\in \eta_{i})+\frac{s(i,j)}{\sum_{r\in \eta_{j}}s(r,j)}\cdot I(i\in \eta_{j}), \end{array}$$

where $s(i,j)=\frac{x_{i}\cdot x_{j}}{{\left|\left|x_{i}\right|\right|}_{2}{\left|\left|x_{j}\right|\right|}_{2}}$ represents the cosine similarity between the features of patients *i* and *j*. Let *η_i_* denote the set of samples which exhibit the top *K* highest similarity with sample *i*. *I*(*cond*) is 1 if *cond* is true, otherwise it is 0. According to a previous research (Rappoport and Shamir 2019), the number of neighbors $K=\frac{\#\mathrm{samples}}{\#\mathrm{clusters}}$; if the number of clusters #clusters is unknown, #clusters is assumed to be 6.

For the *v*th type of omics data, the similarity graph $A^{\left(v\right)}$ and the node feature matrix $X^{\left(v\right)}$ are inputted into the corresponding $GCN^{\left(v\right)}$. The output of *m*th GCN layer is defined as follows:

(2) $$\begin{array}{c} H_{m}^{\left(v\right)}=\sigma \left({\hat{D}}^{{\left(v\right)}^{-\frac{1}{2}}}{\hat{A}}^{\left(v\right)}{\hat{D}}^{{\left(v\right)}^{-\frac{1}{2}}}H_{m-1}^{\left(v\right)}W_{m}^{\left(v\right)}\right). \end{array}$$

Here, ${\hat{A}}^{\left(v\right)}=A^{\left(v\right)}+E$, where *E* is the identity matrix. ${\hat{D}}^{\left(v\right)}$ denotes the node degree matrix of $A^{\left(v\right)}$, which is diagonal. $H_{m-1}^{\left(v\right)}$ denotes the input of the *m*th layer in $GCN^{\left(v\right)}$ and $W_{m}^{\left(v\right)}$ are the model parameters that require training. The function σ(·) represents a nonlinear activation function.

Every GCN of Subtype-MGTP comprises two layers. The processing of the *v*th omics data is denoted as follows:

(3) $$Y^{\left(v\right)}=GCN^{\left(v\right)}(X^{\left(v\right)},A^{\left(v\right)}).$$

Four types of genomics data, serving as the input of the translation module, are fed into corresponding $GCN^{\left(v\right)}$ layers to obtain their intrinsic representations $Y^{\left(v\right)}$. Subsequently, all $Y^{\left(v\right)}$ are fused with weights to obtain the translation result $\hat{P}$, expressed as:

(4) $$\hat{P}=\frac{\sum_{v=1}^{V}Y^{\left(v\right)}}{V}.$$

During the training stage, we only consider samples $X_{\mathrm{part}}$ that have available protein expression data. The collaborative training of all GCNs is guided by the available protein expression data *P*, ensuring that the predicted protein data is as close as possible to the available protein expression data. The loss function is defined as follows:

(5) $$\ell_{t}=\frac{1}{n}||P-\hat{P}|{|}^{2}.$$

where *n* represents the number of training samples. Once training is complete, all parameters of the GCNs are determined. Finally, the genomics data of all samples can be translated into predicted protein data using this trained model.

### 2.3 Deep subspace contrastive learning

To cluster the cancer patients into reliable subtypes based on their predicted protein data, Subtype-MGTP uses a deep subspace contrastive clustering module.

Traditional subspace clustering assumes that data from the same class are distributed within the same subspace, while data from different classes are located in different subspaces (Parsons *et al.* 2004). It suggests that within each class, each sample can be represented as a linear combination of other samples belonging to the same class, a concept referred to as self-expression of the data (Elhamifar and Vidal 2013). Suppose $X=[x_{1},x_{2},\ldots ,x_{N}]\in R^{D\times N}$ denotes a set of *N* samples with *D*-dimension features. The self-expression of data can be simply described as *X* = *XC*, where $C=[c_{1},c_{2},\ldots ,c_{N}]\in R^{N\times N}$ is the self-expression coefficient matrix. Conventional methods for subspace clustering commonly incorporate some regularization terms and minimize self-expression loss to optimize the coefficient matrix *C*, as follows:

(6) $$\begin{array}{cc} & \underset{C}{\min}\;||C|{|}_{q}+\lambda ||X-XC|{|}_{F}^{2}\\ & s.t.\;\mathrm{diag}(C)=0, \end{array}$$

where $||\cdot |{|}_{q}$ denotes a matrix norm, controlling the sparsity of the solution. In this paper, $\ell_{2}$ norm is applied. The constraint on *C* is introduced to prevent trivial solutions, where each data sample is trivially represented by itself. Obviously, it is limited to handling self-expression of data only in linear subspaces.

To capture the intrinsic nonlinearity in omics data, a common strategy is to introduce neural networks before self-expression layer (Ji *et al.* 2017). In this work, we use a symmetric encoding-decoding architecture to achieve nonlinear mapping. Both the encoder and decoder are implemented using GCNs. Given the predicted protein data $P\in R^{D\times N}$ of all samples and the adjacency matrix $A\in R^{N\times N}$, the embedding *Z* (i.e. the output of the encoder) can be formulated as:

(7) Z=GCN(P,A).

The decoder performs the inverse operation of the encoder, enabling unsupervised training to be achieved. Correspondingly, the loss of reconstruction is defined as:

(8) $$L_{\mathrm{rec}}=\frac{1}{N}||P-\hat{P}|{|}^{2},$$

where *N* represents the number of all samples, and $\hat{P}$ denotes the output of the decoder. Notably, although the self-expression layer between the encoder and decoder is a fully connected layer without nonlinear activations and bias, it still achieves nonlinear self-expression of the data, since its input consists of preprocessed nonlinear data from the GCN encoder. Thus, the optimization model in Equation (6) can be transformed to:

(9) $$\begin{array}{cc} & \underset{C,Z}{\min}\;||C|{|}_{q}+\lambda ||Z-ZC|{|}_{F}^{2}\\ & s.t.\;\mathrm{diag}(C)=0. \end{array}$$

To improve the ability of the self-expression layer, we consider introducing contrastive learning, which is one of the state-of-the-art representation learning methods. Specifically, for sample *i*, the representations before and after the self-expression layer are considered as positive pairs, denoted as *z_i_* and $\hat{z_{i}}$, respectively. The rest are treated as negative pairs, represented as *z_i_* and $\hat{z_{j}}$. The contrastive self-expression loss $\ell_{i}$ of *z_i_* is defined as:

(10) $$\begin{array}{c} \ell_{i}=-\text{log}\;\frac{\;\text{exp}(s(z_{i},{\hat{z}}_{i}))}{\sum_{j\neq i}^{N}\;\text{exp}\;(s(z_{i},{\hat{z}}_{j}))}, \end{array}$$

where *s*(*a*, *b*) is the pair-wise cosine similarity. Therefore, the optimization model in Equation (9) can be further transformed to:

(11) $$\begin{array}{cc} & \underset{C,Z}{\min}\;||C|{|}_{q}+\lambda \sum_{i=1}^{N}\ell_{i}\\ & s.t.\;\mathrm{diag}(C)=0. \end{array}$$

By incorporating the loss of reconstruction defined in Equation (8), Equation (11) is refined into a joint optimization problem of deep subspace contrastive self-expression, formulated as follows:

(12) $$\begin{array}{cc} & \underset{C,Z}{\min}\;\lambda_{1}\cdot L_{\mathrm{rec}}+\lambda_{2}\cdot ||C|{|}_{q}+\lambda_{3}\cdot \sum_{i=1}^{N}\ell_{i}\\ & s.t.\;\mathrm{diag}(C)=0, \end{array}$$

where *λ*_1_, *λ*_2_, *λ*_3_ are three trade-off parameters controlling the balance of different losses in training. In our experiments, they were respectively set to 0.01, 1, and 1.

### 2.4 Spectral clustering

After obtaining the subspace representations, the similarity matrix can be calculated as follows:

(13) $$S_{ij}=\frac{1}{2}(|C_{ij}|+|C_{ji}|),$$

where *C* is the aforementioned self-expression coefficient matrix. Let *E* denote the identity matrix. The Laplacian matrix *L* is calculated as follows:

(14) $$L=E-D^{-1/2}SD^{-1/2},$$

where *D* is the corresponding diagonal matrix with $D_{ii}=\sum_{ij}S_{ij}$.

The result of spectral clustering (Ng *et al.* 2001) can be determined by solving the following optimization problem:

(15) $$\begin{array}{cc} & \underset{B}{\min}\;\mathrm{Trace}(B^{T}LB)\\ & s.t.\;B^{T}B=E. \end{array}$$

Here, $B=Y{\left(Y^{T}Y\right)}^{-1/2}$, and $Y={\left[y_{1}^{T},y_{2}^{T},\ldots ,y_{N}^{T}\right]}^{T}$, where *y_i_* denotes the clustering labels, i.e. $y_{i}(k)=1$ indicates that the *i*th patient is classified as the *k*th subtype.

## 3 Results

According to a previous study (Rappoport and Shamir 2018), two standard metrics were used to evaluate the performances of cancer subtyping methods: −log10 *P*-values and the number of significant clinical parameters. The −log10 *P*-value metric is based on the idea that greater differential survival among patients with distinct subtypes indicates a more biologically meaningful clustering outcome. For each cancer dataset, the differential survival −log10 *P*-value is calculated by log-rank test. Enrichment analysis determines the number of significant clinical parameters, encompassing age at diagnosis, gender, pathologic T, pathologic M, pathologic N, and pathologic stage. Among them, discrete parameters are evaluated using the $\chi^{2}$ independence test, while continuous numerical parameters are evaluated using the Kruskal–Wallis test for independence (McKight and Najab 2010). Clinical parameters with adjusted empirical *P*-values below 0.05 are considered enriched within the identified cancer subtypes.

### 3.1 Visualization of the predicted protein data

Due to the high dimensionality of protein data, we used t-SNE (Van der Maaten and Hinton 2008) to visualize distribution differences between predicted and real protein data in a scatter plot. t-SNE was selected for its effectiveness in capturing nonlinear relationships and preserving relative neighborhood relationships, allowing us to explore the local structure and clustering within the dataset. As shown in Supplementary Fig. S1, the predicted data exhibits a similar distribution in the latent space as the real data. It suggests that the multi-omics data translation module faithfully reconstructs the protein data from genomics data.

### 3.2 Comparison with other methods

To assess Subtype-MGTP’s performance in cancer subtype identification, we conducted extensive experiments on the benchmark datasets, comparing it against nine state-of-the-art methods: K-means (Ding and He 2004), LRAcluster (Wu *et al.* 2015), PINS (Nguyen *et al.* 2017), MCCA (Witten and Tibshirani 2009), NEMO (Rappoport and Shamir 2019), Subtype-GAN (Yang *et al.* 2021), Subtype-DCC (Zhao *et al.* 2023), DMCL (Chen *et al.* 2023a), and MOCSS (Chen *et al.* 2023b). To ensure a fair comparison among all methods, a consistent number of clusters was set for each dataset, adhering to established research guidelines (Yang *et al.* 2021). The parameters of the compared methods were configured according to the corresponding papers.

**Figure 2.**
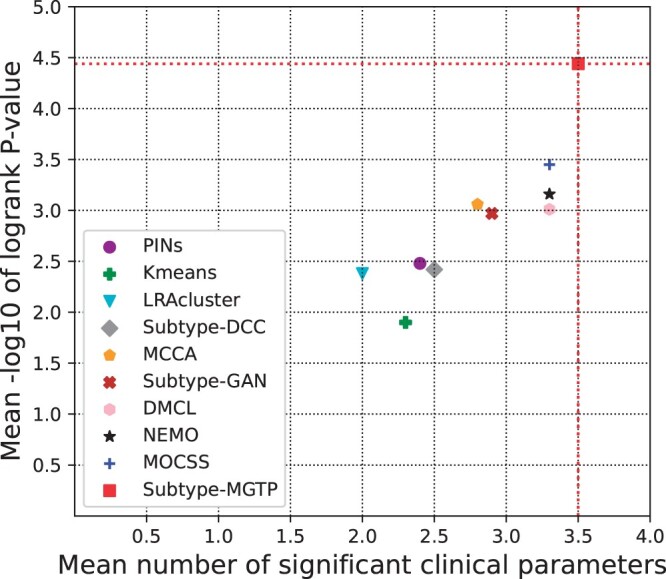
The mean performances of the methods on eight cancer datasets. The *Y*-axis represents the average −log10 logrank test’s *P*-values and the *X*-axis represents the average number of significant clinical parameters in the clusters.

Figure 2 and Table 2 summarize the cancer subtyping performances of the tested methods. Subtype-MGTP surpassed other methods in survival analysis on four datasets, including BLCA, KIRC, SKCM, and STAD. It also exhibited the highest number of significant clinical parameters on five datasets BLCA, BRCA, LUAD, SKCM, and UCEC. Notably, it achieved the best results in both metrics on the BLCA and SKCM datasets. However, its performance on UVM dataset was relatively poor, which we attribute to the small availability of protein data from UVM cancer patients—only 15% of the samples have protein data. Overall, Subtype-MGTP achieved the best performance with an average −log10 *P*-value of 4.44 and an average number of significant clinical parameters of 3.5. Compared to nine alternative methods that solely utilize genomics data, Subtype-MGTP demonstrated outstanding performance, showcasing the effectiveness of its strategy to incorporate proteomics data via multi-omics data translation.

**Table 2.** Performance comparison of the methods.^a^^,^^b^

Method/dataset	BLCA	BRCA	KIRC	LUAD	SKCM	STAD	UCEC	UVM	Mean
Subtype-MGTP	**3.16**/**6**	1.73/**6**	**11.92**/5	2.64/**4**	**5.84**/**3**	**3.48**/3	5.84/**1**	0.92/0	**4.44**/**3.5**
MOCSS	1.83/**6**	1.84/5	6.87/**6**	2.20/3	4.69/**3**	0.09/1	**7.69**/**1**	2.37/**1**	3.45/3.3
NEMO	2.50/**6**	0.98/5	5.68/5	2.36/**4**	4.82/**3**	0.89/1	5.89/**1**	2.15/**1**	3.16/3.3
DMCL	1.91/**6**	0.58/4	7.40/**6**	1.23/3	2.40/**3**	1.59/3	7.05/**1**	1.92/0	3.01/3.3
MCCA	1.26/4	**2.60**/5	9.80/3	0.92/**4**	0.75/2	1.09/2	5.34/**1**	2.71/**1**	3.06/2.8
Subtype-GAN	0.83/5	1.39/5	6.08/**6**	**3.67**/3	0.98/2	0.47/1	7.15/**1**	**3.13**/0	2.97/2.9
Subtype-DCC	2.09/5	1.17/4	5.13/4	1.84/2	1.49/**3**	1.22/1	4.42/**1**	1.98/0	2.42/2.5
PINs	2.52/5	1.18/2	4.29/**6**	3.03/0	0.35/1	2.38/**4**	3.69/**1**	2.41/0	2.48/2.4
LRAcluster	0.21/1	0.16/5	7.26/**6**	0.17/1	1.86/1	0.28/1	6.60/**1**	2.52/0	2.38/2.0
K-means	0.41/2	0.06/4	5.73/**6**	0.31/1	1.02/2	0.10/2	5.86/**1**	1.67/0	1.90/2.3

a A/B denotes −log10 *P*-values/number of significant clinical parameters.

b Bold indicates the best performance, while underline indicates a suboptimal performance.

Details of the clustering results for all methods are provided in Supplementary Tables S1 and S2 due to space limitations. In addition, Supplementary Fig. S2 offers a visual representation of the distribution of cluster sizes for Subtype-MGTP. To visually highlight the differences among cancer subtypes, the survival curves of the subtypes identified by Subtype-MGTP on each dataset are illustrated in Supplementary Fig. S3. As time progresses, the divergence in survival curves becomes more prominent, suggesting distinct survival probabilities among the subtypes. This indicates that Subtype-MGTP is capable of identifying patient populations with different prognoses.

### 3.3 Analysis of effectiveness and stability

**Figure 3.**
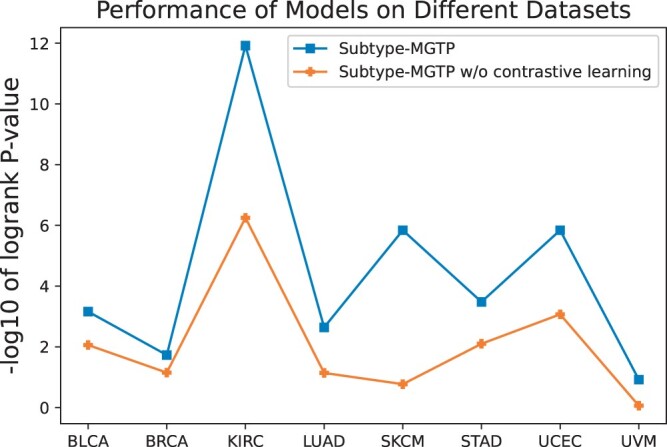
Performance comparison of Subtype-MGTP and its variant without contrastive learning.

To explore the impact of incorporating contrastive learning into deep subspace clustering, we conducted ablation experiments and illustrated the result in Fig. 3. It is evident that the performance significantly decreased after removing contrastive learning in the self-expression layer, indicating its integral role in our model.

**Figure 4.**
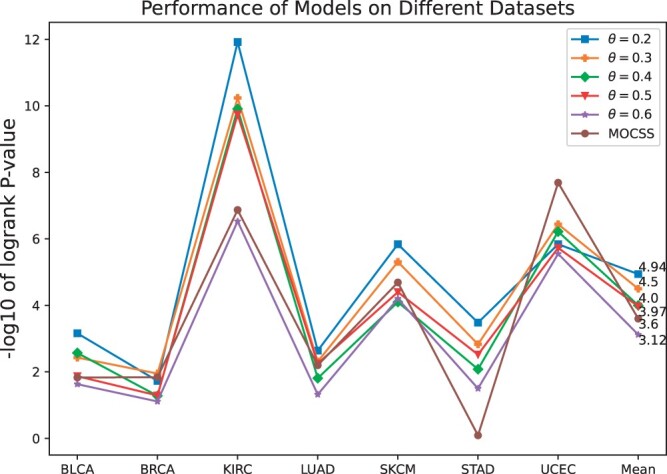
Performance comparison of Subtype-MGTP on simulated data with varying protein data missing rates.

In addition, to further explore the capabilities and limitations of Subtype-MGTP, we conducted simulation experiments with varying missing rates of protein expression data. In the original dataset, the overall missing rate is approximately 0.2. Starting from a missing rate of 0.3, we incrementally increased the missing rate by 0.1 on each dataset (excluding UVM, which already exhibited a high missing rate of up to 85%). Figure 4 illustrates the performance of Subtype-MGTP under different missing rates, along with the strongest baseline model, MOCSS, for comparison. The detailed data is listed in Supplementary Table S3. The results indicate that a missing rate of 0.5 is the maximum acceptable limit for Subtype-MGTP. When the missing rate exceeds 0.5, the model’s performance significantly deteriorates and falls below the strongest baseline in most cases.

**Figure 5.**
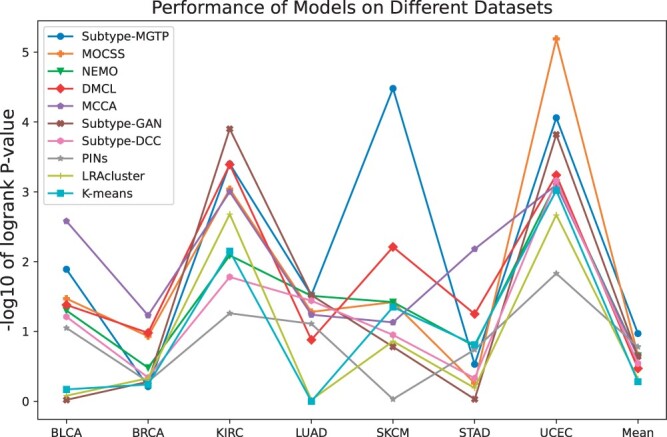
Performance comparison of the methods in subtyping patients without protein expression data when the data missing rate θ=0.5.

Although predicted protein data are utilized in clustering for all patients, it is still necessary to investigate whether the clustering performance of patients with missing protein expression data is improved. Therefore, when the missing rate was set to 0.5 (excluding the UVM dataset), we picked out the patients without protein expression data for subtyping and subsequently performed survival analysis. Figure 5 presents the survival analysis results for various methods on seven datasets, and the clustering sizes are reported in Supplementary Tables S4 and S5. Overall, Subtype-MGTP achieved good performance even with a missing rate of 0.5. Despite poor outcomes observed in several datasets (BRCA, STAD), it ranked first in terms of average performance. This demonstrates the effectiveness of Subtype-MGTP in clustering patients without protein expression data.

**Figure 6.**
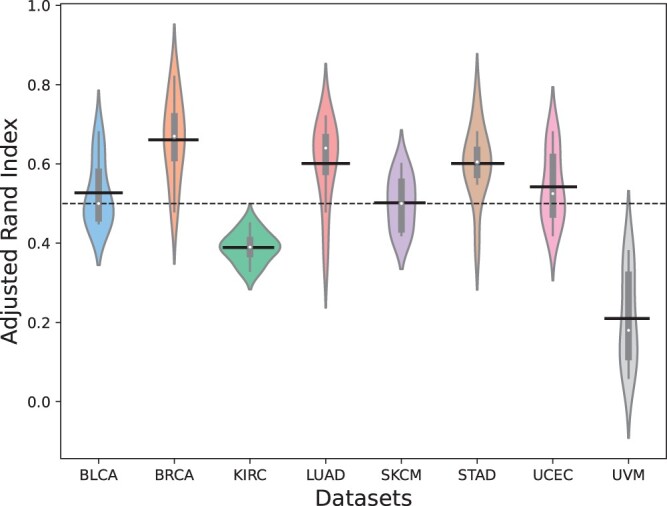
The distribution of ARI on different datasets.

Finally, we followed the approach outlined in (Von Luxburg *et al.* 2010) to assess the stability of our clustering results. Under the original data missing rate, for each predicted protein dataset, we conducted ten rounds of random sampling, selecting 80% of the data each time. The subsample was then clustered using the deep subspace contrastive clustering module, and we compared the clustering results of each subsample with the original clustering results. The Adjusted Rand Index (ARI) (Hubert and Arabie 1985) was utilized to measure the similarity between two different clustering results, ranging from −1 to 1: 1 indicating perfect agreement, 0 suggesting independence, and values close to −1 indicating significant disagreement. As illustrated in Fig. 6, the clustering results of Subtype-MGTP on most datasets exhibit high stability with a high ARI range of 0.5 to 1. However, the clustering results on the UVM dataset exhibit relative randomness with a low ARI. We attribute this to the high missing rate of the UVM dataset, resulting in the failure of translation and the inability to obtain meaningful features for clustering. Consequently, we conclude that the clustering of Subtype-MGTP is stable when the translation module is effectively functioning. Supplementary Table S6 provides the detailed ARI for each test.

### 3.4 Case study

In our experiments, the SKCM dataset stood out as one on which Subtype-MGTP demonstrated excellent overall performance. Subtype-MGTP clustered the patients of the dataset into four subtypes, as depicted in the survival curves presented in the SKCM subfigure of Supplementary Fig. S3. These curves indicate that subtype 1 and subtype 4 have poorer prognoses, while subtype 3 exhibits the best prognoses. The t-SNE visualization of these subtypes presented in Supplementary Fig. S4A reveals distinct spatial distributions: most patients of subtypes 1 and 4 are located on the upper-half part of the subfigure, whereas most patients of subtype 3 are positioned at the right-bottom corner, and those of subtype 2 are predominantly situated in the middle-bottom part.

**Figure 7.**
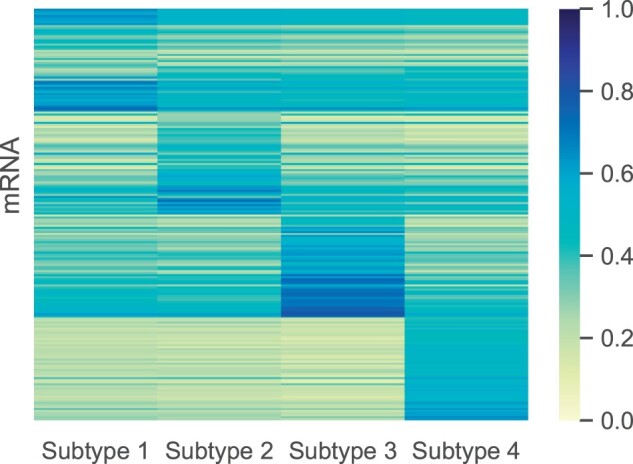
Differentially expressed mRNAs of the SKCM subtypes.

**Figure 8.**
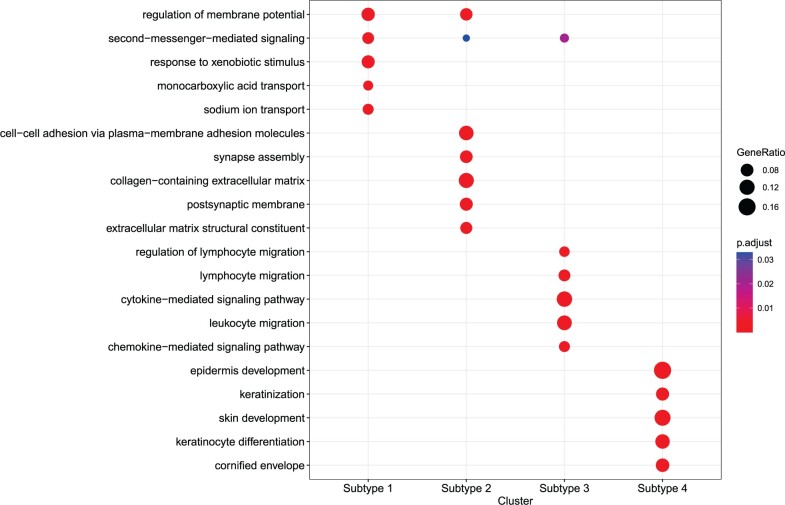
GO enrichment analysis of the differentially expressed genes of the four SKCM subtypes.

For the dataset, ANOVA F-values were calculated for each gene to analyze if there were significant differences in mRNA expression among different subtypes. Multiple-testing adjustments were conducted using FDR controlling procedures. After calculating differentially expressed genes (DEGs), we analyzed the molecular level differences among the four subtypes. Figure 7 presents a heatmap of the top 50 DEGs of each SKCM subtype. Notably, each subtype showed significantly differential expression, indicating that these subtypes may involve different molecular mechanisms. Particularly, we used t-SNE to visualize the most promising potential biomarkers for each subtype, i.e. “HNF1A,” “TMEM100,” “C10orf105,” and “ODZ2,” as depicted in Supplementary Fig. S4. We observed that the potential biomarkers exhibit relatively high expression in their respective clusters (indicated in red) and relatively low expression in other clusters (indicated in blue). This highlights a strong and interpretable association between the biomarkers and the identified subtypes. Furthermore, gene ontology (GO) annotations for each set of DEGs were conducted using the R package “clusterProfiler” (Yu *et al.* 2012). Figure 8 illustrates the GO pathways enriched by the DEGs of four subtypes, which are relevant to the initiation, progression, and metastasis of SKCM. The enrichment analysis demonstrates that DEGs in different subtypes are associated with distinct biological pathways in SKCM.

Overall, the results of the case study in SKCM further demonstrate the biological significance of the subtypes identified by our model.

## 4 Conclusion

Cancer subtype identification plays a crucial role in precision medicine, enabling the development of targeted treatment strategies and improving the chances of survival for cancer patients. The availability of vast multi-omics datasets has facilitated the development of computational models for cancer subtyping. This paper presents a novel integrated framework called Subtype-MGTP, which utilizes multi-omics data translation and deep subspace contrastive clustering to fulfill the task. Specifically, our model uses partial protein data as guidance to translate multiple genomics data into protein data. Subsequently, based on the predicted protein data, it uses a deep subspace contrastive clustering method to obtain distinct subtypes of the specific cancer. As indicated by the experimental results, Subtype-MGTP exhibited excellent performance compared to nine state-of-the-art methods on broadly used benchmark datasets. Extensive simulation experiments with various data missing rates demonstrated the robustness and effectiveness of Subtype-MGTP. It also exhibited stability in clustering analysis, provided that effective translation is ensured. Finally, we conducted a case study on the SKCM dataset, where promising biomarkers were identified for each subtype, and differentially expressed genes were found enriched in distinct pathways, indicating differences in the formation of subtypes.

The effectiveness of Subtype-MGTP can be attributed to two main reasons. Firstly, proteomics data may offer more valuable insights into cancer subtyping compared to genomics data. By translating multiple genomics datasets into proteomics data, Subtype-MGTP bridges the gap between genotype and phenotype, enhancing our understanding of cancer biology. Secondly, the integration of contrastive learning into deep subspace clustering enables Subtype-MGTP to thoroughly explore the relationships between different samples before and after the self-expression layer. This enhancement significantly improves the data mining performance of multi-omics data, resulting in biologically more distinguishable cancer subtypes.

However, it’s important to acknowledge the associations between genetic mutations and molecular mechanisms of cancer, which facilitate the discovery of biomarkers related to specific subtypes and deepen our understanding of cancer’s molecular mechanisms of cancer. Yet, the high dimensionality of genomic mutation data poses a significant challenge to its integration into our study. Effectively integrating various types of omics data still remains a crucial research area for the future.

Overall, Subtype-MGTP exhibits unique advantages in integrating multi-omics data with imbalanced data sizes, contributing to its effectiveness in cancer subtype identification. We anticipate that our work will provide a new perspective for multi-omics data integration studies.

## Supplementary Material

btae360_Supplementary_Data

## Data Availability

The data used in this study are available in the source code repository.
