# Supplementary material for: Subtype-MGTP: a cancer subtype identification framework based on multi-omics translation
Source: Bioinformatics. 2024 Jun 10;40(6):btae360. doi: 10.1093/bioinformatics/btae360 (PMC11194476; doi:10.1093/bioinformatics/btae360)
Supplement: btae360_Supplementary_Data [file btae360_supplementary_data.pdf]

Supplementary Materials for "Subtype-MGTP: A Cancer Subtype Identification Framework Based on Multi-Omics Translation"

June 1, 2024

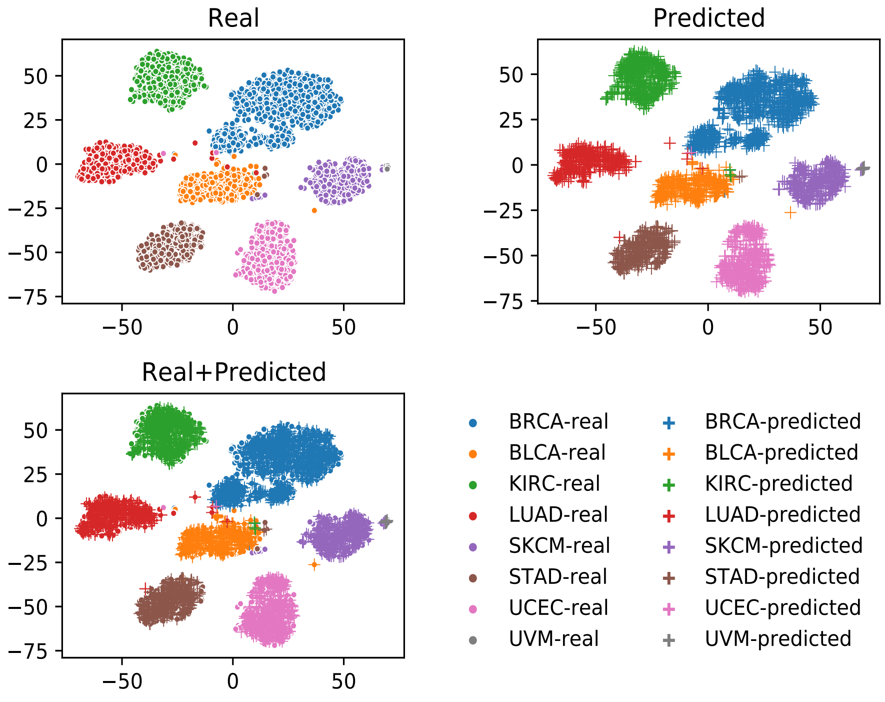

Figure S1: The scatter plots of both real and predicted protein data. Samples of distinct tumor subtypes are in different colors, while real data and predicted data are marked with different symbols.

Table S1: The sizes of subtypes clustered by the methods on the following datasets: BLCA, BRCA, KIRC, and LUAD.

Method	Dataset			
	BLCA	BRCA	KIRC	LUAD
Subtype-MGTP	68/152/47/60/72	192/292/155/211/181	95/128/157/108	133/156/201
NEMO	90/64/103/64/78	238/173/185/211/224	169/233/16/70	191/130/169
MOCSS	85/82/79/63/90	123/365/226/142/175	135/40/195/118	116/216/158
DMCL	85/96/81/55/82	229/118/177/248/259	157/156/80/95	159/154/177
MCCA	90/64/103/64/78	238/173/185/211/224	169/233/16/70	191/130/169
Subtype-GAN	76/79/106/64/74	243/216/227/103/242	92/119/80/137	66/147/110
Subtype-DCC	331/51	370/91/336/234	3/465/20	109/381
K-means	63/104/73/84/75	107/193/271/286/174	70/157/173/88	116/193/181
PINs	181/87/131	286/165/460/39/32	194/98/196	130/170/190
LRAcluster	59/83/64/88/105	278/192/202/179/180	108/168/146/66	202/153/135

Table S2: The sizes of subtypes clustered by the methods on the following datasets: SKCM, STAD, UCEC, and UVM.

Method	Dataset			
	SKCM	STAD	UCEC	UVM
Subtype-MGTP	148/123/109/66	172/160/75	38/236/138/98	38/8/9/25
NEMO	157/121/99/69	96/175/136	109/98/131/172	23/16/19/22
MOCSS	107/100/120/119	35/205/167	122/52/243/93	42/19/16/3
DMCL	147/125/57/117	156/146/105	199/62/159/90	33/19/19/9
MCCA	157/121/99/69	96/175/136	109/98/131/172	23/16/19/22
Subtype-GAN	94/125/77/52	102/83/98	80/144/96/65	16/25/14/10
Subtype-DCC	86/121/130/109	25/265/117	101/231/178	6/56/18
K-means	167/110/87/82	169/97/141	188/98/81/143	23/14/19/24
PINs	232/214/	194/112/39/62	100/118/79/89/124	52/28
LRAcluster	141/85/93/127	74/167/166	146/202/74/88	22/17/19/22

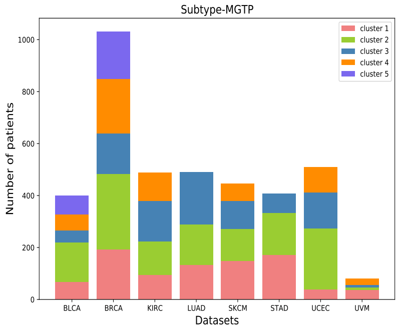

Figure S2: The distribution of subtype sizes clustered by Subtype-MGTP on each dataset.

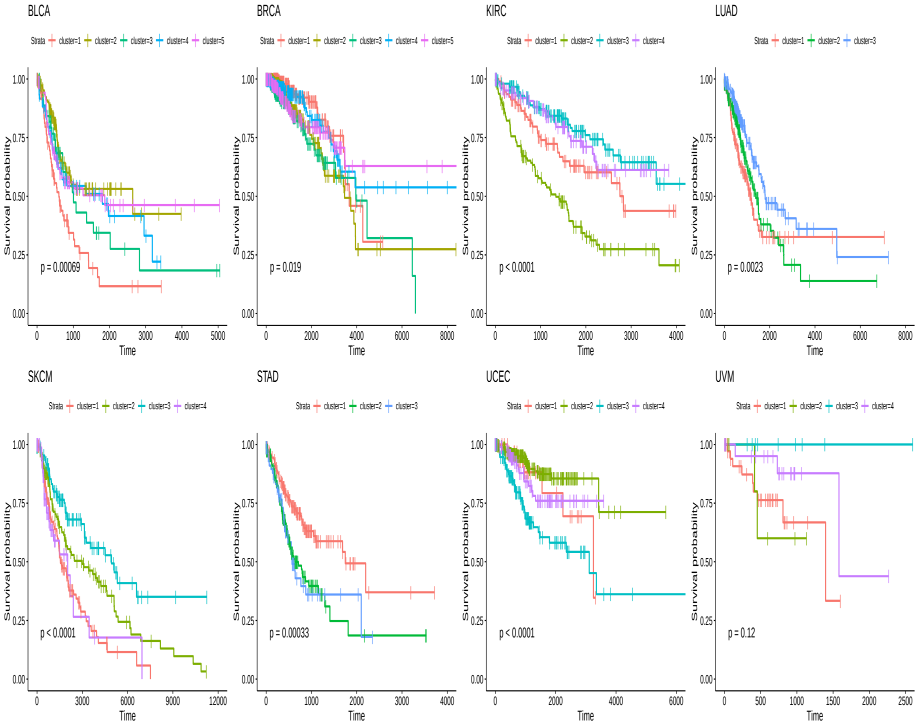

Figure S3: Survival analysis curves of the subtypes clustered by Subtype-MGTP on eight datasets. The different colors represent different cancer subtypes.

Table S3: Performance evaluation of Subtype-MGTP in simulation experiments with varied missing rates of real protein data.

Missing rate	BLCA	BRCA	KIRC	LUAD	SKCM	STAD	UCEC	Mean
$\theta = 0.2$	3.16/6	1.73/6	11.92/5	2.64/4	5.84/3	3.48/3	5.84/1	4.94/4.0
$\theta = 0.3$	2.43/5	1.95/6	10.24/5	2.31/4	5.31/3	2.83/1	6.44/1	4.50/3.5
$\theta = 0.4$	2.57/5	1.28/6	9.91/6	1.81/4	4.10/3	2.82/0	6.22/1	4.00/3.5
$\theta = 0.5$	1.87/5	1.29/6	9.74/6	2.25/3	4.40/2	2.52/1	5.73/1	3.97/3.4
$\theta = 0.6$	1.63/5	1.11/6	6.53/5	1.32/2	4.20/3	1.50/1	5.55/1	3.12/3.3

[1] A/B denotes -log10 P-values/the number of significant clinical parameters.

Table S4: At a missing rate of 0.5, the size of clusters of various methods on the following datasets: BLCA, BRCA, KIRC, and LUAD.

Method	Dataset			
	BLCA	BRCA	KIRC	LUAD
Subtype-MGTP	91/14/41/15/39	164/91/79/76/106	63/96/17/68	107/46/92
NEMO	40/36/52/29/43	118/94/88/105/111	86/112/10/36	98/55/92
MOCSS	43/41/38/34/44	61/188/103/69/95	68/22/94/60	55/110/80
DMCL	46/58/34/27/35	119/48/95/113/141	81/80/34/49	72/79/94
MCCA	97/4/16/42/41	153/62/100/145/56	51/80/12/101	76/75/94
Subtype-GAN	33/37/57/34/39	119/107/114/47/129	44/59/43/64	33/81/46
Subtype-DCC	161/27	182/47/170/117	2/234/8	45/200
K-means	33/56/39/37/35	54/99/144/136/83	27/81/90/46	52/103/90
PINs	92/51/57	143/88/227/21/13	86/49/109	64/88/93
LRAcluster	29/46/28/47/50	147/90/105/93/81	57/85/77/25	96/80/69

Table S5: At a missing rate of 0.5, the size of clusters of various methods on the following datasets: SKCM, STAD and UCEC.

Method	Dataset		
	SKCM	STAD	UCEC
Subtype-MGTP	72/60/40/51	69/84/51	77/75/38/65
NEMO	80/59/55/29	52/86/66	56/46/67/86
MOCSS	43/60/64/56	13/93/98	66/29/110/50
DMCL	71/64/33/55	79/72/53	102/29/84/40
MCCA	14/55/32/122	14/91/99	60/72/73/50
Subtype-GAN	51/63/42/23	47/42/55	39/78/41/35
Subtype-DCC	37/62/68/56	14/129/61	51/110/94
K-means	81/57/38/47	77/54/73	90/45/46/74
PINs	115/108	98/48/22/36	62/63/31/38/61
LRAcluster	67/50/43/63	43/77/84	79/95/40/41

Table S6: The Adjusted Rand Index between the clusterings of subsample and the clusterings of original dataset.

Dataset	Rounds									
	1	2	3	4	5	6	7	8	9	10
BLCA	0.51	0.52	0.45	0.6	0.45	0.46	0.68	0.47	0.49	0.64
BRCA	0.73	0.61	0.53	0.66	0.68	0.78	0.69	0.63	0.82	0.48
KIRC	0.39	0.4	0.38	0.41	0.45	0.33	0.37	0.34	0.43	0.39
LUAD	0.67	0.67	0.48	0.72	0.66	0.58	0.37	0.66	0.58	0.62
SKCM	0.58	0.56	0.42	0.42	0.54	0.5	0.42	0.6	0.48	0.5
STAD	0.58	0.55	0.62	0.62	0.4	0.59	0.76	0.57	0.68	0.64
UCEC	0.68	0.55	0.47	0.42	0.48	0.66	0.5	0.55	0.47	0.64
UVM	0.21	0.37	0.11	0.38	0.1	0.15	0.12	0.26	0.34	0.06

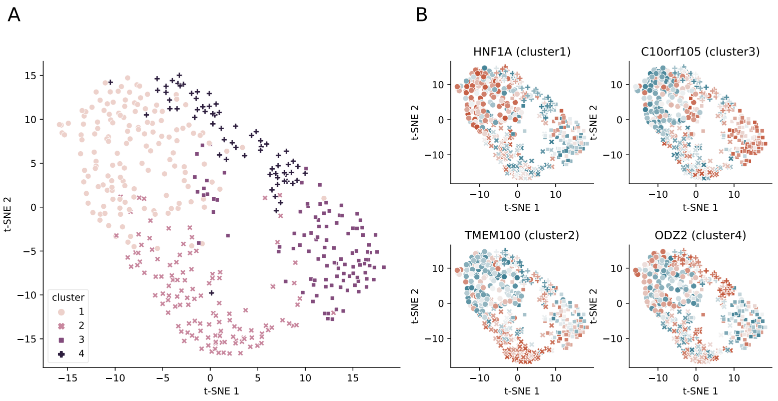

Figure S4: SKCM subtypes and biomarkers. (A) the t-SNE visualization of SKCM subtypes. (B) the expression of biomarker mRNAs across all samples; The colors gradually transition from deep blue to deep red, indicating an increase in gene expression.
